# Polygenic Associations between Motor Behaviour, Neuromotor Traits, and Active Music Engagement in Four Cohorts

**DOI:** 10.1101/2025.03.27.645667

**Published:** 2025-03-30

**Authors:** T.L. Henechowicz, P.L. Coleman, D.E. Gustavson, Y.N. Mekki, S. Nayak, R. Nitin, A.C. Scartozzi, E.S. Tio, R van Klei, D. Felsky, M.H. Thaut, R.L. Gordon

**Affiliations:** 1. Music and Health Science Research Collaboratory, Faculty of Music, University of Toronto; 2. Krembil Centre for Neuroinformatics, Centre for Addiction and Mental Health; 3. Vanderbilt Genetics Institute, Vanderbilt University Medical Center; 4. Music Cognition Laboratory, Department of Otolaryngology–Head and Neck Surgery, Vanderbilt University Medical Center; 5. Center for Digital Genomic Medicine, Vanderbilt University Medical Center; 6. Institute for Behavioral Genetics, University of Colorado Boulder; 7. Department of Psychiatry, University of Toronto; 8. Division of Biostatistics, Dalla Lana School of Public Health, University of Toronto; 9. Rotman Research Institute, Baycrest Hospital, Toronto, ON; 10. Department of Anthropology, University of Toronto; 11. Temerty Faculty of Medicine, University of Toronto; 12. Vanderbilt Brain Institute, Vanderbilt University; 13. Department of Psychology, Vanderbilt University

**Keywords:** musicality, music engagement, music training, CLSA, WLS, BioVU, polygenic scores, genetic correlation, motor system, brain structure, gold-MSI, musical achievement

## Abstract

Phenotypic investigations have shown that actively engaging with music, i.e., playing a musical instrument or singing may be protective of motor decline in aging. For example, music training associated with enhanced sensorimotor skills accompanied by changes in brain structure and function. Although it is possible that the benefits of active music engagement “transfer” to benefits in the motor domain, it is also possible that the genetic architecture of motor behaviour and the motor system structure may influence active music engagement. This study investigated whether polygenic scores (PGS) for five behavioural motor traits, 12 neuromotor structural brain traits, and seven rates of change in brain structure traits trained from existing discovery genome-wide association studies (GWAS) predict active music engagement outcomes in four independent cohorts of unrelated individuals of European ancestry: the Canadian Longitudinal Study on Aging (CLSA; N=22,198), Wisconsin Longitudinal Study (WLS; N=4,605), Vanderbilt’s BioVU Repository (BioVU; N=6,150), and Vanderbilt’s Online Musicality study (OM; N=1,559). Results were meta-analyzed for each PGS main effect across outcomes and cohorts, revealing that PGS for a faster walking pace was associated with higher amounts of active music engagement. Within CLSA, a higher PGS for walking pace was associated with greater odds of engaging with music. Findings suggest a shared genetic architecture between motor function and active music engagement. Future intervention-based research should consider the genetic underpinnings of motor behavior when evaluating the effects of music engagement on motor function across the lifespan.

## 1. Introduction

Actively engaging with music, i.e., playing a musical instrument or singing, is an underappreciated lifestyle factor in healthy ageing because it is a cognitively stimulating, involves motor learning, coordination, and multisensory integration (1). Research suggests that skills gained from playing instruments or singing may “transfer” to enhanced skills in a non-related domain (e.g., cognitive or motor skills) and changes in brain structure and function (1-3). Motor function is an integral part of neurocognitive health in aging, where motor decline precedes cognitive decline (4,5). There are several lines of phenotypic evidence showing how active music engagement may be protective of motor function. For example, a neuroimaging meta-analysis showed that musicians, compared to non-musicians, have greater auditory-motor network connectivity and structural adaptations in regions important for motor control including the corpus callosum, internal capsule, and sensorimotor and subcortical areas (6). Behaviourally, musicians, compared to non-musicians, have enhanced musical motor skills (e.g., audio-motor synchronization) (7-9), better performance on standardized motor function assessments (10,11), faster reaction time in spatial (12), multisensory integration (13), and visuomotor tasks (14), and enhanced motor sequence learning and retention (15,16). Music training interventions may also heighten motor skill development in children and adolescents (17,18). Given what is now known about genetic contributions to brain development and plasticity (19-21), it is possible that these “transfer” effects are partially influenced by shared genetic variation (22). The current investigation seeks to understand how genetic factors for motor behaviour and neuromotor traits may influence individual differences in music engagement.

Given the clear behavioural and neural links between musical instrument training and the motor system, several authors hypothesize that long-term or regular playing of an instrument or singing is likely an environmental factor that modulates motor system neural plasticity (23). In parallel, prior individual differences in motor skills or neuromotor features may contribute to who seeks out musical training (2,3,24), and prior work does not rule out the possibility that alternative or additional shared biology, i.e., shared genetic architecture, underlies these phenotypic correlations. For example, the potential “transfer” of childhood musical instrument engagement to verbal ability four years later in adolescence is in part driven by shared genetic effects (25). To investigate the hypothesized shared genetic architecture between active music engagement and motor phenotypes, we can adapt insights from a recent musicality cross-trait framework, the *Musical Abilities, Pleiotropy, Language, and Environment* (MAPLE) (22). The MAPLE framework makes a case that both musical abilities and musical environments may arise from genetic effects, i.e., the niche-picking phenomenon where individuals are drawn to environmental influences for which they have higher genetic liability to well adapted. Second, commonly found phenotypic associations between musicality and motor traits can be explained through partially shared genetic architecture and mediating neurobiological phenotypes (22).

Indeed, active music engagement and motor traits are moderate to highly heritable. Music-related traits have an average heritability of 42% (26), including 78% for musical instrument engagement (25), 33–66% for musical aptitude (27), and 41–69% for musical practice (28,29). Recent genomic studies show evidence for SNP-based heritability of 12% for music engagement (30). For motor traits, a twin study found evidence for 68% and 70% heritability of motor control and motor learning, respectively (31). Additionally, genetic studies of neurobiological endophenotypes, e.g., the thickness, volume, and change in volume/thickness of motor system brain structures, are available publicly through *Enhancing Neuroimaging Genetics through Meta-analysis Consortiums’* GWASs and show evidence for the heritability and detectable polygenic signals (19,32,33). Accordingly, cross-trait population-level genomic methods, i.e., polygenic scores, can leverage existing GWASs for behavioural motor and neuromotor phenotypes to understand their shared biological etiology with active music engagement.

In this pre-registered study (https://doi.org/10.17605/OSF.IO/SQ2NC), we examined whether PGSs for three categories of motor traits—behavioural, brain structure, and rate of change of brain structure—predict active music engagement in four cohorts: the Canadian Longitudinal Study on Aging (CLSA) (34,35), Vanderbilt’s BioVU repository (BioVU) (36,37), Wisconsin Longitudinal Study (WLS) (38), and the Vanderbilt’s Online Musicality Study (OM) (30). We predicted that greater genetic predisposition for motor function (i.e., greater PGS for walking pace, lower PGS for reaction time, lower PGS for hand muscle weakness, lower PGS for clinical motor diagnosis of Parkinson’s disease, and lower PGS for Developmental Coordination Disorder risk) would each be associated with higher amounts of music engagement. For neuromotor phenotypes, we predicted associations between genetic predispositions for brain structure and its rates of change in brain structure with active music engagement, without specific directional hypotheses.

## 2. Methods

### 2.1. Data

#### Study populations (target cohorts)

Four large cohort studies were analyzed: CLSA (N=22,198), WLS (N=4,605), BioVU (N=6,150), and OM (N=1,559). Given the limited data availability in these cohorts, analyses were constrained to individuals of European ancestry (see **Supplementary Methods 1.1-1.4** for phenotyping and quality control protocols). We extracted active music engagement phenotypes and categorized the outcomes into constructs including music practice, music achievement, and music engagement (see **Supplementary Table 1.** and **Supplementary Methods 1.1-1.4**).

#### Discovery GWAS summary statistics used for PGS construction

PGSs were constructed using summary statistics from published GWASs of motor behaviour traits (5 traits), structural motor brain traits (12 traits), and the rate of change of brain structure across the lifespan (19) (7 traits) (see **Appendix B. Supplementary Table 2.** for descriptions of the discovery GWAS). All discovery GWAS samples were non-overlapping with target cohorts and were of European ancestry to match the target cohorts’ ancestries to avoid population stratification issues(39).

#### Motor behaviour discovery GWAS

We selected motor behaviour traits from the GWAS atlas (https://atlas.ctglab.nl/), catalogue (https://www.ebi.ac.uk/gwas/), and from a general literature search. Available GWASs included reaction time(40), self-reported walking pace (41), hand muscle weakness (grip strength <30 kg in males and <20 kg in females). (42), motor coordination difficulties in children (43), and Parkinson’s disease case status (44) (see **Appendix B. Supplementary Table 2.**).

#### Neuromotor brain structure discovery GWAS

We selected recent neuroimaging GWASs from the ENIGMA consortium for PGS of structural brain traits (32,33). For subcortical regions, we selected four of seven structures (32), the nucleus accumbens, pallidum, putamen, and caudate, because of their importance for motor learning and music training (6,45). For cortical structures, we extracted Grasby et al.’s meta-GWASs (33), specifically, we selected overall cortical thickness and six cortical thickness phenotypes from regions essential to motor and sensory systems: the precentral, postcentral, inferior parietal, insula, middle temporal, and superior temporal gyri. We also included Tissink et al.’s GWAS of combined cerebellar grey matter and cerebellar white matter volume (46).

#### Neuromotor rate of change discovery GWAS

Lastly, we included Brouwer et al.’s meta-GWAS of change rates in brain structure (19). We selected a subset of brain regions and phenotypes that matched our cross-sectional analyses and had SNP-based heritability greater than 0, i.e., the total brain volume, total cerebellar white matter volume, total cortical volume, total cortical thickness, pallidum volume, putamen volume, and nucleus accumbens volume. Rate-of-change PGSs were calculated using meta-GWAS summary statistics, which meta-analyzed SNP effects independent of age differences between cohorts. Therefore, positive PGSs represent the genetic liability for positive change (more growth or less shrinkage) across the lifespan, and negative PGSs represent negative change (less growth or more shrinkage) across the lifespan.

#### PGS construction

PGSs were calculated using PRS-CS or PRS-CS-auto, which use a Bayesian regression framework and place a continuous shrinkage prior on SNP effect sizes (47), outperforming traditional clumping and thresholding methods (48). We used PRS-CS-auto to construct PGSs for the GWAS summary statistics with N>200,000 and PRS-CS with phi=0.01 for smaller sample sizes (i.e., neuroimaging and motor coordination GWASs) as suggested for highly polygenic traits (47). All PGSs were calculated with default parameters, a=1 and b=0.5 (47), and with the 1000 Genomes Project Phase 3 European linkage disequilibrium (LD) reference panel (47,49).

### 2.2. Statistical Analysis

Statistical analyses were performed using R statistical software v4.0.2. Linear models were used to test for the main effects of each motor PGS on all music engagement outcomes within each cohort. For each outcome, 24 models were fit, each including one of the 24 motor PGSs with covariates of 10 genomic principal components, age, and biological sex, yielding 168 models. Continuous and binary outcomes were modelled with ordinary least squares linear and logistic regression, respectively. As pre-registered, multiple testing corrections were applied to PGS main effect *p*-values for the total number of models within each cohort (e.g., OM has 4 outcomes × 24 models for 72 tests; WLS has 2 outcomes × 24 models for 48 tests; BioVU has 1 outcome × 24 models for 24 tests; CLSA has 1 outcome × 24 models for 24 tests). Multiple test correction was assessed using the Benjamini-Hochberg false discovery rate (FDR) procedure, with a corrected *p*-value (*q*FDR) of *q*FDR<0.05 indicating a significant effect. We assessed model fit indices of *R^2^* for continuous outcomes and Nagelkerke-Pseudo-*R^2^* and *AUC* for binary outcomes. To evaluate out-of-sample performance and overfitting, we performed resampling analyses using 100 iterations of the .632 bootstrapping method commonly applied in GWAS (50,51). In secondary analyses, we fitted the same 168 models with PGS-by-sex interactions, applying the same multiple-test correction approach. Influential observations were identified using Cook's distance (threshold of > 4/N-*k*-1), and we conducted sensitivity analyses by removing influential observations.

Lastly, we conducted meta-analyses of the effect sizes for each PGS association across cohorts. Since outcomes were continuous and dichotomous, we converted log odds ratios to standard mean differences prior to meta-analysis (52). Inverse-variance weighted random mixed-effect meta-analyses were conducted using the restricted maximum likelihood estimator with 150 maximum iterations from the *metafor* package in R (53). We applied FDR multiple testing correction for the 24 meta-analyses.

## 3. Results

### 3.1. Descriptive Statistics

**Figure 1.**
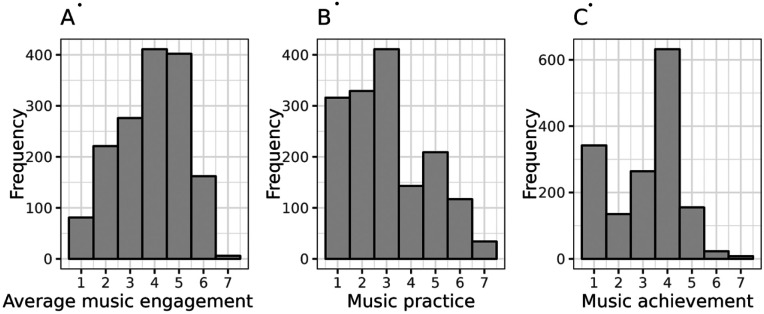
Distributions of Continuous Outcomes from Vanderbilt’s Online Musicality Study (OM). *Note.* (A) the distribution of average music engagement score, (B) music practice (as defined by question 33 in the gold-MSI), and music achievement (as defined by the creative achievement questionnaire). See **Supplementary Methods 1.4** for further phenotyping definitions in the OM cohort. Participants’ mean average score for music engagement was 3.99±1.36, and the median was 4.25 (IQR=3.25,5.25). The median self-report music practice hours was 3, corresponding to 1 hour per day (IQR=2,4). The median of self-report music achievement was 4, corresponding to “I have played or sung, or my music has been played in public concerts in my hometown, but I have not been paid for this” (IQR=3,5). All three music engagement phenotypes were significantly positively correlated: *r_s_*=0.75 (*p*<0.001) for average music engagement and music practice, *r*_s =_ 0.84 (*p*<0.001) for average music engagement and music achievement, and *r*_s_=0.58 (*p*<0.001) for music achievement and music practice.

Table 1 has descriptive statistics for N=22,198 unrelated individuals from CLSA (62.99±10.15 years old, 50% female, 77.55% had completed a post-secondary degree/diploma), N=4,605 from WLS (64.22±2.50 years old, 51% female, and the average education was 13.89±2.40 years), N=6150 from BioVU (53.13±16.38 years old, 41% female, mean Area Deprivation Index of 0.33±0.12), and N=1559 from OM (45.85±16.33 years old, 74% female, 79.74% had at least a bachelor’s degree or equivalent). Distributions of the continuous outcomes in OM are in Figure 1. For case-control cohorts, we compared the available socio-economic and education variables (See Note in Table 1). See **Supplementary Methods 1.1-1.4.** for inclusion/exclusion criteria and **Figures S1A-S1D** for correlations between age, sex, and PGSs within each cohort.

### 3.2. Meta-analyses of PGS Models

**Figure 2.**
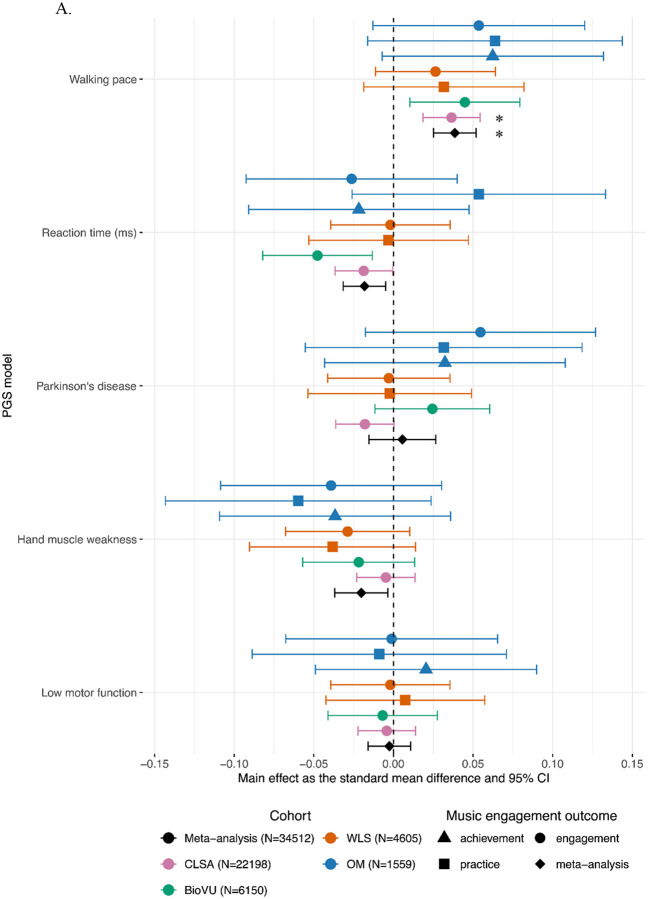

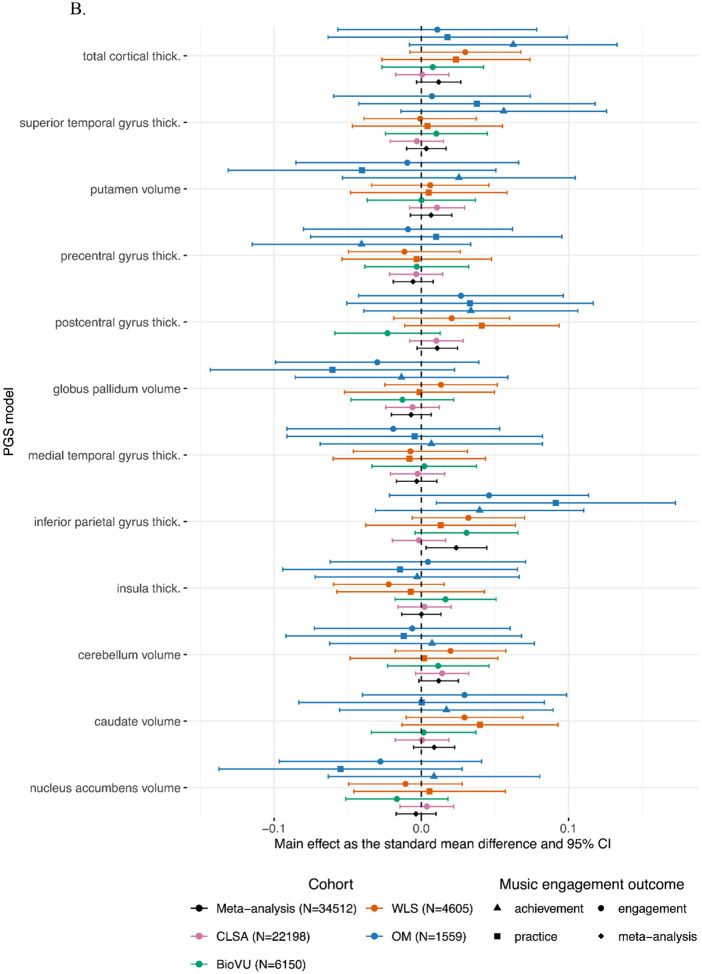

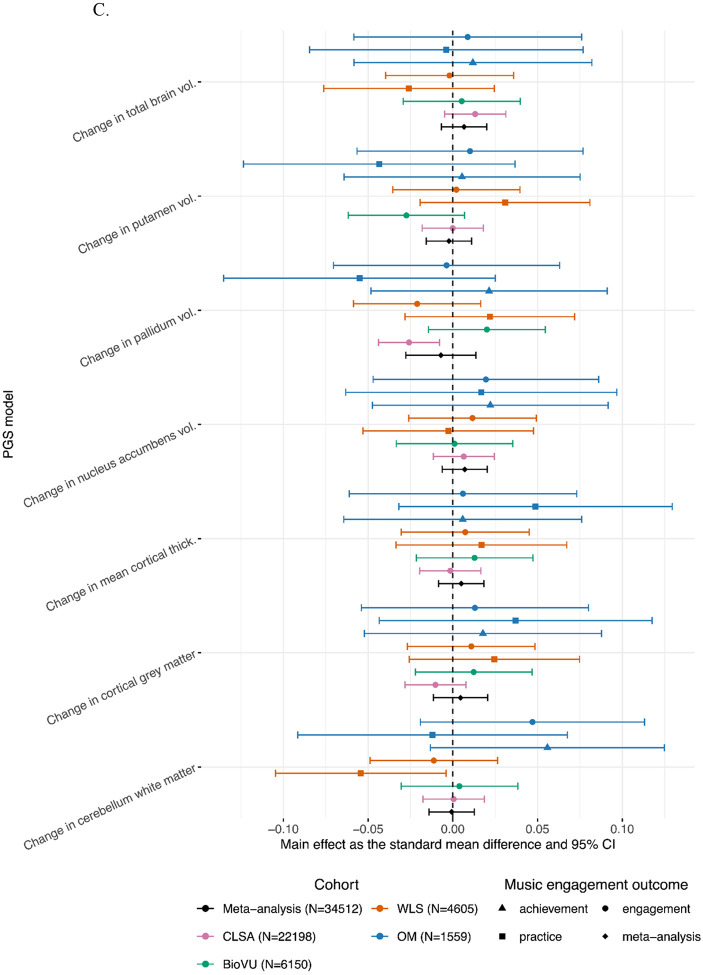
Forest Plot of 24 PGS Main Effects and Meta-analyses Across Outcomes

The meta-analyzed PGS for walking pace was positively associated with active music engagement (*b=*0.04, 95% CI [0.03, 0.05], *p*<0.001, *q*FDR<0.001) with no significant heterogeneity among studies (*Q*(6)=1.68, *p*=0.95). At an uncorrected threshold of *p*<0.05, PGS for reaction time was negatively associated with active music engagement (*b*=−0.02, 95% CI [−0.03, −0.005], *p*=0.008, *q*FDR=0.09) with no significant heterogeneity among studies (*Q*(6)=7.10, *p*=0.31) and PGS for hand muscle weakness was negatively associated with active music engagement (*b*=−0.02, 95% CI [−0.04, −0.003], *p*=0.02, *q*FDR=0.14) with no significant heterogeneity among studies (*Q*(6)=4.46, *p*=0.61) (See Figure 2A). Additionally, PGS for inferior parietal gyrus thickness was positively associated with active music engagement (*b=*0.02, 95% CI [0.003, 0.04], *p*=0.02, *q*FDR=0.14) with no significant heterogeneity among studies (*Q*(6)=9.44, *p*=0.15) (See Figure 2B).

### 3.3. Single PGS Model Results

#### Main Effects of Motor Behaviour PGSs on Music Engagement Outcomes

PGS for walking pace was positively associated with greater odds of music engagement only in CLSA (OR =1.07, 95% CI [1.03, 1.10], *p*<0.001, *q*FDR=0.002) (see Figure 2A). At an uncorrected threshold of *p*<0.05, PGS for walking pace was associated with greater odds of music engagement in BioVU (OR=1.08, 95% CI [1.02, 1.15], *p*=0.01, *q*FDR=0.13). At an uncorrected threshold of *p*<0.05, there was suggestive evidence that higher PGS for reaction time (i.e., slower reaction time) was associated with lower odds of music engagement in CLSA (OR=0.97, 95% CI [0.94, 1.00], *p*=0.04, *q*FDR=0.33) and in BioVU (OR=0.92, 95% CI [0.86, 0.98], *p*=0.007, *q*FDR=0.13). See **Supplementary Table 3** for all main effects and boot-strapped performance indices and **Supplementary Table 4** for sensitivity analysis results.

#### Main Effects of Structural Brain PGSs on Music Engagement Outcomes

At an uncorrected threshold of *p*<0.05, PGS for inferior parietal gyrus thickness was positively associated with music practice in OM (*b=*0.09, 95% CI [0.01, 0.17], *p*=0.03, *q*FDR=0.93) (See Figure 2B).

#### Main effects of Neuromotor Rate of Change PGSs on Music Engagement Outcomes

At an uncorrected threshold of *p*<0.05, there were potential associations between neuromotor rate of change PGSs and music engagement, although there was no consistency across cohorts (See Figure 2C). In CLSA, PGS for the rate of change in pallidum volume was negatively associated with odds of being a musically active case (OR=0.95, 95% CI [0.92, 0.99], *p=*0.005, *q*FDR=0.06), suggesting that engaging with music is associated with a genetic predisposition for more shrinkage of the pallidum. In WLS, PGS for the rate of change in cerebellum white matter volume was associated with decreased odds of practicing a musical instrument (OR=0.91, 95% CI [0.83, 0.99], *p=*0.03, *q*FDR=0.90), i.e., greater likelihood of music practice was associated with a higher genetic predisposition for more shrinkage of the cerebellum white matter.

#### Interaction Effects of Sex on PGS Models

There were no significant sex-by-PGS interactions after multiple testing corrections. However, at the suggestive *p<*0.05 threshold, there were some potential interaction effects of sex and PGS for walking pace (See **Supplementary Table 5** and **Figures S2–9**).

All effects represented in a forest plot as effect sizes and 95% confidence intervals. Meta-analyzed effects are represented as diamonds. For continuous outcomes, main effects were estimated using linear regression and are represented as an effect size. For case-control or dichotomized outcomes, logistic regression models were used to calculate log odds, which were then transformed into standard mean differences. The results are panelled by PGS category (a) motor behaviour (5 PGSs), (b) neuromotor brain structure (12 PGSs), and (c) neuromotor rate of change in regional volume/thicknesses (7 PGSs). Asterisks (*) denote significant meta-effects after multiple-test correction (*q*FDR*<*0.05). See **Supplementary Table 1.** for cohort information and **Supplementary Table 2.** for GWAS discovery source studies. **Supplementary Table 6.** has source data for plotting.

## 4. Discussion

Our investigation revealed insights into the potential shared genetic relationship between music engagement and motor traits. First, the genetic predisposition for enhanced motor function, i.e., higher PGS for walking pace, was significantly associated with greater music engagement in CLSA and once meta-analyzed across four cohorts. We also observed potential associations between greater PGSs for inferior parietal gyrus thickness, lower PGSs for muscle weakness, and lower PGSs for reaction time and more active music engagement. Despite this, the meta-analyses and cohort-level analyses provide inconclusive evidence as to whether PGSs for neuromotor traits predict active music engagement.

The meta-analyses revealed that the PGS for walking pace was positively associated with active music engagement, with the strongest effect in CLSA. The genetics of walking pace are important for motor function and are connected to several domains of health. For example, faster self-reported walking is causally associated with lower cardiac and stroke risk (41,56) and genetically correlated with lower cardiometabolic, respiratory, psychiatric, and all-cause mortality risk and higher educational attainment (41,57). We observed patterns showing that genetic associations between walking pace and active music engagement may differ between males and females, which is expected, given the role of sex differences in brain development and aging (58-60). The meta-analyses across cohorts also revealed patterns of association between PGS for muscle weakness and active music engagement. Hand muscle weakness, measured by low hand grip strength, is a simple motor performance metric that provides a window into neural resources needed to commit a motor action, e.g., hand grip strength recruits corticospinal tract motor neurons (61). Hand grip strength also has genetic associations with frailty, cardiac, psychiatric, motor, and general health traits (62-66). Lastly, we also observed trends of associations with PGS for inferior parietal gyrus thickness and active music engagement. The inferior parietal lobe is critical for music processing because of its role in sensorimotor integration (Sun et al., 2022) and musical rhythm processing (68,69). Despite the potential relationship between PGS for inferior parietal gyrus thickness and music engagement, we did not observe significant meta-analyzed or cohort-level associations with other neuromotor traits.

Our results provide evidence for potential shared genetics between active music engagement motor traits, suggesting that investigations of “transfer” from musical to non-musical skills should consider individual differences. Several authors have suggested that genetic factors and gene-by-environment interactions likely confound the literature on the “transfer” to non-musical domains (3,3,24,70,71). Schellenberg and colleagues have evaluated the state of the literature on music-based “transfer” to non-musical domains of cognition, suggesting that music-induced benefits are weak correlations (3,71,72). Individual differences (not limited to genetic predispositions) possibly magnify the observed correlations between music engagement and other domains (71). Thus, gene-by-environment interactions can be incorporated into future music-based intervention studies to improve their rigour.

Our results suggest that PGSs for the rate of change in brain structure in motor regions do not account for differences in active musical engagement. However, Brouwer et al.’s age-independent meta-GWASs on the rate of change of brain structures capture the average rate of change across the lifespan rather than during specific developmental or aging periods. Moreover, our findings do not rule out the possibility that other plasticity traits may influence active music engagement. For example, the neural structure of white matter tracts, especially the right corticospinal tract organization, in infanthood predicts rhythmic and musical abilities in school-age children (73). Future investigations should also consider functional neuromotor phenotypes, e.g., the genetic architecture of functional brain networks (74), especially given the relevance of sensorimotor or audiomotor networks to active music engagement (6).

Although the meta-analyses revealed promising insights, we discuss some study limitations. While BioVU and OM had validated outcomes, the outcomes in WLS and CLSA had unknown psychometric profiles. The phenotypes also included singing together with musical instrument engagement, which may require different neuromotor resources, e.g. singing requires coordination of laryngeal, oropharyngeal, and facial muscles from the corticobulbar tract (75) and musical instruments require fine motor control of the upper limbs that descend from the corticospinal tract (2). Therefore, motor PGSs and active music engagement associations may be deflated due to this heterogeneity. Current initiatives, i.e., the *Musicality Genomics Consortium,* are tackling this issue by validating succinct measurements to implement in biobanks.

Second, the predictive power of PGSs are constrained by discovery and target cohort sample sizes and phenotype measurements (39), e.g., the childhood motor functioning and neuromotor discovery GWASs had smaller sample sizes. Additionally, current motor behaviour GWASs do not capture the full range of complex motor phenotypes, but recent studies show the feasibility of collecting motor learning data from thousands of individuals online (76) for future GWASs. Lastly, due to data availability, our primary analyses were constrained to genetic similarity with European ancestry due to differences in allele frequencies and the limited generalizability of PGSs across ancestries (77,78). Future work should leverage multi-ancestry methods and focus on musicality phenotyping in diverse populations.

## 5. Conclusion

PGS for higher self-reported walking pace may be associated with greater music engagement. However, analyses did not yield sufficient evidence to support that PGSs for neuromotor phenotypes predict active music engagement. Our results suggest that shared genetic factors for motor function may predict active music engagement, holding significance for longitudinal studies and interventions aiming to understand the transfer of musical learning to non-musical domains.

## Figures and Tables

**Table 1.** Descriptive Statistics for Target Cohorts

*Canadian Longitudinal Study on Aging*				
Characteristic	*n* for non- missing	Overall Sample, N=22,198	Several times a year or more frequently, N=4,556	Once a year or less, N=17,642
		*n (%)*	*n (%)*	*n (%)*
*Sex* [self-report]	22,198			
Female		11,153 (50.24%)	2,219 (48.71%)	8,934 (50.64%)
Male		11,045 (49.76%)	2,337 (51.30%)	8,708 (49.36%)
*Education* *	22,161			
Less than secondary school graduation		1,171 (5.28%)	152 (3.39%)	1,019 (5.79%)
Secondary school graduation, no post-secondary		2,127 (9.60%)	264 (5.80%)	1,863 (10.58%)
Some post-secondary education		1,677 (7.57%)	280 (6.15%)	1,397 (7.93%)
Post-secondary degree/diploma		17,186 (77.55%)	3,856 (85.71%)	1,330 (75.70%)
		M (SD)	M (SD)	M (SD)
*Age* [years]	22,198	62.99 (10.15)	62.21 (10.08)	63.18 (10.16)

*Wisconsin Longitudinal Study*				
Characteristic	*n* for non- missing	Overall Sample, N=4,605	Played a musical instrument at 35 often or sometimes N=1,130	Played a musical instrument at 35 never N=3,475
		*n (%)*	*n (%)*	*n (%)*
*Sex* [self-report]	4,605			
Female		2,348 (51.00%)	720 (63.72%)	1,628 (46.85%)
Male		2,257 (49.02%)	410 (36.28%)	1,847 (53.15%)
		M (SD)	M (SD)	M (SD)
*Age* [years]	4,605	64.22 (2.50)	64.00 (2.32)	64.24 (2.55)
*Education* [years]*	4,592	13.69 (2.40)	14.45 (2.53)	13.71 (2.34)

*Vanderbilt ‘s BioVU Repository*				
Characteristic	*n* for non- missing	Overall Sample, N=6,150	Musically active patient cases, N=1,259	Population- matched controls, N=1,491
		*n (%)*	*n (%)*	*n (%)*
*Gender* [self-report]	6,150			
Female		2,515 (41%)	553 (44%)	1,962 (40%)
Male		3,635 (59%)	706 (56%)	2,929 (60%)
		M (SD)	M (SD)	M (SD)
*Age* [median age at record in years]	6,150	53.13 (16.38)	53.07 (16.52)	53.15 (16.34)
*Area Deprivation Index* †*	5,265	0.33 (0.12)	0.31 (0.12)	0.33 (0.12)

*Vanderbilt’s Online Musicality Study*		
Characteristic	*n* for non-missing	European subset N=1,559
		*n (%)*
*Gender* [self-report]	1,559	
Female		1,146 (73.51 %)
Male		413 (26.49%)
*Education*	1,555	
less than high school		2 (0.13%)
high school (or equivalent)		46 (2.96%)
some college or associate’s degree		267 (17.17%)
bachelor’s or equivalent		553 (35.56%)
master’s or equivalent		510 (32.80%)
doctorate or equivalent		177 (11.38%)
		M (SD)
*Age* [years]	1,559	45.85 (16.33)

*Note.* *Denotes significant differences in SES/education variables between cases and controls. In CLSA, musically engaged cases showed significantly greater education levels (X^2^ (3, N=22161) = 180.17, *p* < .001). In WLS, those who played a musical instrument “sometimes” or “often” at the age of 35 years old had more years of education compared to those who “never” played (t(1796.3) = −8.7152, *p* < .001). In BioVU, musically active cases had a lower Area Deprivation Index than controls (t(1602.8) = 5.23, p < .001, 95% CI [0.01, 0.03]), indicating potential evidence for less socio-economic deprivation, although a nominal difference (54). *†*The Area Deprivation Index is a ranking of neighbourhoods by socioeconomic conditions in the United States that accounts for multiple domains of factors, including income, education, employment, and housing quality, to inform health delivery and policy (54,55).
